# ‘I’ve put diabetes completely on the shelf till the mental stuff is in place’. How patients with doctor-assessed impaired self-care perceive disease, self-care, and support from general practitioners. A qualitative study

**DOI:** 10.1080/02813432.2018.1487436

**Published:** 2018-06-22

**Authors:** Mads Aage Toft Kristensen, Ann Dorrit Guassora, Anne Beiter Arreskov, Frans Boch Waldorff, Bibi Hølge-Hazelton

**Affiliations:** a Department of Public Health, The Research Unit for General Practice and Section of General Practice, University of Copenhagen, Copenhagen, Denmark;; b Southern Køge Medical Centre, Køge, Denmark;; c Research Unit of General Practice, Institute of Public Health, University of Southern Denmark, Odense, Denmark;; d Zealand University Hospital, Roskilde, Denmark;; e Department of Regional Health Research, University of Southern Denmark, Odense, Denmark

**Keywords:** Multimorbidity, chronic disease, patient acceptance of health care, general practice, self-care, continuity of patient care

## Abstract

**Objective:** This paper investigated patients’ experiences of disease and self-care as well as perceptions of the general practitioner’s role in supporting patients with impaired self-care ability.

**Design:** Qualitative interviews with 13 patients with type 2 diabetes, concurrent chronic diseases, and impaired self-care ability assessed by a general practitioner. We analyzed our data using systematic text condensation. The shifting perspectives model of chronic illness formed the theoretical background for the study.

**Results:** Although most patients experienced challenges in adhering to recommended self-care activities, many had developed additional, personal self-care routines that increased wellbeing. Some patients were conscious of self-care trade-offs, including patients with concurrent mental disorders who were much more attentive to their mental disorder than their somatic diseases. Patients’ perspectives on diseases could shift over time and were dominated by emotional considerations such as insisting on leading a normal life or struggling with limitations caused by disease. Most patients found support in the ongoing relationship with the same general practitioner, who was valued as a companion or appreciated as a trustworthy health informant.

**Conclusion:** Patient experiences of self-care may collide with what general practitioners find appropriate in a medical regimen. Health professionals should be aware of patients’ prominent and shifting considerations about the emotional aspects of disease. Patients valued the general practitioner’s role in self-care support, primarily through the long-term doctor-patient relationship. Therefore, relational continuity should be prioritized in chronic care, especially for patients with impaired self-care ability who often have a highly complex disease burden and situational context.

**Key points**
  Little is known about the perspectives of disease and self-care in patients with a doctor-assessed impaired ability of self-care.
  • Although patients knew the prescribed regimen they often prioritized self-care routines that increased well-being at the cost of medical recommendations.
  • Shifting emotional aspects were prominent in patients’ considerations of disease and sustained GPs’ use of a patient-centred clinical method when discussing self-care.
  • Relational continuity with general practitioners was a highly valued support and should be prioritized for patients with impaired self-care.

## Introduction

Patient self-care is a central theme in the worldwide reorganization of chronic care through disease management programs (DMPs) [1], which aim to improve the management of chronic diseases and reduce health care utilization [2]. In the last decade, DMPs have been implemented in Denmark and require general practitioners (GPs) to stratify the patients’ need for specialist care and self-care support based on assessments of self-care ability and disease severity (Table 1) [3]. Guidelines are available for assessment of the latter but not for the former.

**Table 1.** An example of how GPs are expected to stratify patients with type 2 diabetes determining the level of chronic care.

		Disease regulation	
		Well	Poor
Self-care	High	I	III
		General practice	General practice
			Specialist care
	Low	II	IV
		General practice	General practice
		Self-care support	Specialist care
			Self-care support

However, bringing self-care considerations into the management of chronic care can be challenging. An English study of clinical encounters in general practice showed that self-care topics were infrequently part of the consultations because both the GPs and patients with chronic diseases experienced difficulties in addressing self-care topics without upsetting the doctor-patient relationship [4]. GPs valued patient involvement in health care, but also experienced conflict between the focus on self-management and other areas of professional responsibility such as prioritizing the biomedical aspects of care at the expense of exploring the patient’s perspective [5]. Patients often found themselves in practical and moral dilemmas related to their performance of self-care as prescribed [6] giving rise to issues of honour and shame [7].

The concept of self-care has evolved over time and now many different definitions exist [8]. A recent systematic review highlighted the need for further clarification of the concept of self-care for clinical practice and research and suggested a comprehensive and operational definition (Box 1) [9].

Box 1 Self-care involves a range of care activities deliberately engaged throughout life to promote physical, mental and emotional health, maintain life and prevent disease. Self-care is performed by the individual on their own behalf, for their families, or communities, and includes care by others. In the event of injury, disability or disease, the individual continues to engage in self-care, either on their own or in collaboration with healthcare professionals. Self-care includes social support and the meeting of social and psychological needs. Self-care provides the continuity of care between interactions with the healthcare system, enabling individuals to manage their disease or disability and maintain well-being.

The Danish DMPs describe self-care as ways to maintain health, to obtain quality of life, and to be responsible for treatment of chronic diseases [2]. Reviews about how medical papers used the concept of self-care found that health professionals and authorities referred to self-care in a rather narrow context related to disease [10], whereas patients were unfamiliar with the term and rarely distinguished health-related behaviours from other aspects of their lives [11]. Now that self-care has become central in chronic care, it is important to explore further patient perspectives on self-care [12].

Patients with type 2 diabetes (T2DM) are usually managed in general practice and should be referred to specialist care in cases of increasing disease severity (Table 1). However, it is also the case that patients with less severe T2DM might receive specialist care, while others with severe T2DM remain in general practice [13,14]. Some of these patients with severe T2DM are in an exposed position with concurrent chronic diseases and complex psychosocial needs, which is known to affect diabetes care negatively [15]. From the GP’s perspective, these challenges can reveal themselves in a lowered capacity for self-care in terms of difficulty in adhering to the regimen recommended by treatment guidelines [16]. The GPs may often tailor treatment for these patients, but little is known about perceptions of disease and self-care in patients whose GPs have assessed their self-care ability as impaired.

Hence, the aim of this paper is to investigate experiences of disease and self-care in patients with GP-assessed impaired self-care ability, who have been diagnosed with T2DM and other concurrent diseases. A secondary aim is to explore how these patients perceive the GP’s role in the support of self-care.

## Methods

In Denmark, almost the entire population is registered with a GP for primary health care, which is free at the point of use, with limited co-payment for medications and visits to, e.g. a physiotherapist or a psychologist. As most patients with chronic conditions are treated in general practice and can only consult specialists upon referral, Danish GPs have a central position in the assessment of patients’ self-care ability [2], which forms part of the decision-making process to refer patients to other health professionals.

This study is linked to a larger study in general practices located in two economically disadvantaged, rural municipalities in Southeastern Denmark [17]. Initially, 12 GPs identified 36 patients with high disease complexity in terms of T2DM, one or more other chronic diseases and impaired self-care ability (group IV of stratification in Table 1). To ensure clinical relevance, patients’ impaired self-care was pragmatically defined as the GP’s assessment of difficulty in following the recommended treatment in general practice or in the hospital. The GPs identified 31 patients eligible for participation. Upon purposive sampling to gain maximal variation in gender, age, and comorbidities, 18 patients were invited, of whom 15 agreed to participate in our study. Interviews were cancelled by two patients due to exacerbation of disease. The first author, MATK, conducted individual, semi-structured interviews with participants in 2015. An interview guide provided a flexible framework for questioning within the following areas: living with multiple chronic conditions; experiences of following the regimens at home; and collaborating with the GP, the hospital or the community. The participants knew MATK’s background as a GP. All but one interview took place in the patients’ homes and lasted between 50 and 80 minutes each.

The interviews were audiotaped and transcribed verbatim. We used systematic text condensation in the analysis of data. The procedure consists of the following steps: (1) total impression – from chaos to themes; (2) identifying and sorting meaning units – from themes to codes; (3) condensation – from code to meaning; (4) synthesis – from condensation to descriptions and concepts [18]. The research team included medical doctors, with and without GP training, and a nurse all of whom assisted MATK with the initial identification of themes. Analysis was primarily performed by MATK together with researchers BBH and ADG who mentored the process and control-coded eight interviews. Open coding by hand was used to analyze the transcripts and through comparison of these codes, the coding framework was negotiated among the researchers. Meaning units were organized in documents by code groups and condensations of code groups were written. Table 2 provides an example of the analysis.

**Table 2.** shows the process for analyzing our data using systematic text condensation.

Theme	Meaning units (relevant quotes)	Condensation	Synthesis
Understanding the significance of severe mental disorders in combination with diabetes and other somatic conditions.	*My biggest wish is to get my nut under control. I can live with my diabetes and the pain in my leg, but getting rid of these bad thoughts would broaden my horizons and help me to relax in what I do (Patient 4).*	Relief from mental symptoms is prioritized first	Severe mental disorders overrule attention to somatic conditions.
	*My mind takes up most space, while the diabetes is easy to forget… This night, I didn’t sleep. Then I spend all day speculating about the past (Patient 9).*	Mental symptoms are ever present	
	*My back takes up much space, when it hurts and the mental stuff takes up extremely much space… We have heard so much about the diabetes, but I have put it completely on the shelf until the mental stuff is in place, because if I cannot survive mentally, then it doesn’t bloody matter how much diabetes I’ve got (Patient 10).*	Somatic conditions can be overshadowed by mental disorders.	
	*It hurts me that neither my family, the GP nor the nurses have taken my anxiety seriously. They probably think that I use it as an excuse for being allowed to smoke. But it certainly is not; these attacks are very, very serious. When I get them, I must be alone and I think so much about why I have to live like this (Patient 12).*	Mental symptoms can be neglected by the surrounding network and thereby block the care of somatic conditions.	

**Figure 1.**
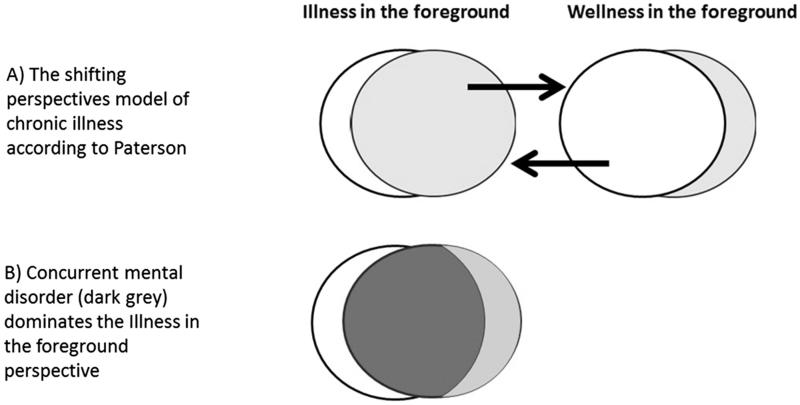
The shifting perspectives model of chronic illness with modification.

During analysis, The shifting perspectives model of chronic illness was chosen among other theories to form the study’s theoretical frame of reference (Figure 1(A)). In this model, the patient experience of chronic diseases tend to shift between the focused perspectives of illness and wellness [19]. Variations during the course of disease can induce a shift in perspective, but social circumstances often have a great influence as well.

## Results

In addition to long-term T2DM, often complicated by diabetic foot ulcers or neuropathy, the 13 patients we interviewed had common chronic conditions and a majority had mental disorders or addiction problems adding to their complexity (Table 3). The findings from our analysis of the patient interviews were sorted into three major groups: perceptions of self-care, disease, and the GP’s support of self-care (Box 2).

Box 2 Overview of findings

**Perceptions of self-care**

Personal self-care routines

1. Personal self-care activities

2. Personal dietary habits

Challenges of self-care related to the medical regimen

1. Compromised habits and preferences

2. Keeping up joy to avoid total failure

3. Other somatic diseases

4. Mental disorders rule

**Perceptions of disease**

1. Coming to terms or struggling with disease

2. Fearing the next complication

3. The small bright spots

4. Being done with the future

**Perceptions of the GP’s support of self-care**

1. The GP as a companion or a guardian

2. The GP as a health consultant

3. The GP has no supportive role

**Table 3.** Shows the profiles of the interviewed patients and the GP-identified case patients.

	Interview sample *n* = 13	All case patients *n* = 36
Age, years		
Mean	59.2	62.5
Range	37–72	37–81
Gender, number (percentage)		
Male	8 (62%)	21 (58%)
Female	5 (38%)	15 (42%)
Chronic Conditions, number (percentage)		
Diabetes	13 (100%)	36 (100%)
Heart disease	6 (46%)	18 (50%)
Mental disorder	5 (38%)	16 (44%)
Obesity	5 (38%)	14 (39%)
Addiction	5 (38%)	9 (25%)
Musculoskeletal disorders	4 (31%)	8 (22%)
Respiratory disease	1 (8%)	4 (11%)

### Perceptions of self-care

#### Personal self-care routines

All patients were familiar with the medical prescriptions such as medications, regular physical activities and diabetic diet, but most patients expressed difficulty in adopting the recommended life style. All patients explained that they had developed additional everyday routines resulting in increased wellbeing or reduced symptoms, but did not necessarily improve disease regulation. These routines were grouped as personal self-care activities and personal dietary habits.

***Personal self-care activities*** could be a walk with the dog, going for a swim or gardening. These single physical activities were primarily related to the patient’s immediate wellbeing. Some patients with mental disorders had sedentary strategies to reduce stress and find mental ease, for instance playing a computer game or smoking cannabis.

Sometimes I smoke a joint… and it does not help the diabetes, because you eat more, but it helps my mind… It makes me indifferent to everything (Patient 9).

***Personal dietary habits*** were adopted by most patients. Some of them were in line with the medical recommendations while others were solely based on personal preferences or experiences. One patient was convinced that the intake of ginger juice had improved her blood sugar significantly, while another found that his blood sugar did not change with the consumption of beer and continued this habit despite medical advice.

If I felt bad in the morning, I would drink an ice cold coca-cola with sugar. After a quarter, I would feel bloody well… The dietitian said that it was not allowed and ‘oh, it is dangerous’. But it worked for me (Patient 1).

### Challenges of self-care related to the medical regimen

The recommended self-care activities could be challenged by symptoms and the practical obstacles of diseases, the patient’s subjective preferences or the patient’s available mental resources.

#### Treatment compromises habits and preferences

A considerable number of patients could not adopt all recommended life style changes because they conflicted with their existing and valued habits. Some of these patients were open with their GP about making a reflected choice to hold on to their habits and rely on the medication.

My GP tells me to exercise, but I tell her that I can’t… I don’t care (about exercise)… I would hundred times rather sit in my chair by the television or read… No, I don’t bother to start walking (Patient 3).

Other patients experienced their attempts to perform the recommended life style changes as an ongoing struggle.

I am not allowed to eat any fruit at all, because it increases the sugar value… I try … but it is damned difficult when you like apples, bananas and oranges (Patient 5).

#### Keeping up joy to avoid total failure

Some patients had experienced periods of depression without any joy of living. It could be due to their social circumstances, such as a divorce, but it also related to facing the facts of disease and the potential nearness of death. To avoid giving up the zest for life, a large number of patients said, that they had decided not to think or talk about their disease, even though disease had a significant impact on their daily lives. Instead, they tried to look on the bright side in an effort to obtain the best quality from the life that remained to them.

You have to look at it from the humorous side… You must keep some joy and laugh sometimes, otherwise it will all fail… and then I would not be able to manage my situation (Patient 8)

One patient was afraid of falling into a hole of dark thoughts and giving up with few resources left for managing life, where the meaning of self-care would decrease with the meaning of life. Another patient remembered such a period:

Some years ago… I was so depressed (due to a failed marriage)… Then I did not care a shit about my diabetes or anything, to say it in plain Danish (Patient 6).

These patients had typically shifted their perspective from a life with disease in the foreground to a life with diseases in the background. They focused on wellness, and in this space self-care tasks disturbed the everyday lives much less.

I do not think much of disease anymore. In the morning, I say to myself: ‘Today is a good day!’ It may rain or the sun may shine… I try to enjoy the remains of my life as much as possible… (Patient 7).

#### Other somatic diseases

The patients generally experienced that other somatic diseases made up permanent or temporary barriers to self-care activities for diabetes. Many patients were confused about the recommended diet because of other complicating factors such as intake of vitamin K-anticoagulants, which had additional limitations that could make dietary changes difficult to carry through.

Because I take anticoagulants, I am not allowed to eat the recommended diet for diabetes… No cabbage – cauliflower and so on… They tell me to eat much corn and peas, which I don’t like. It is really difficult for me (Patient 2).

Fatigue or physical discomfort could drain patients’ energy preventing them from performing self-care activities or going to health care appointments. Some days, patients stayed in bed or limited their activities. Other patients could not exercise as recommended due to breathlessness or physical limitations, e.g. amputation. Some patients said that health professionals did not respect their limitations or even grasp their severity.

I take morphine daily for my back… and when you get too obese, you get even more back pain… it’s a vicious spiral… Unintelligent persons might say: ‘you can just go for a run or ride your bike’. No, I can’t, because then I cannot walk for ten days owing to the back (Patient 10).

#### Mental disorders rule

Three patients had severe mental disorders requiring psychiatric treatment, and they emphasized that these symptoms, if left untreated, overruled their attention to diabetes and other somatic conditions. Attempts to get relief from mental symptoms, like consumption of alcohol, could increase indifference to self-care. These patients understood such trade-offs and their consequences. Mental disorders simply overshadowed somatic diseases if thoughts and sleep were constantly disturbed.

We have heard so much about the diabetes, but I’ve put it completely on the shelf till the mental stuff is in place, because if I can’t survive mentally, then it doesn’t bloody matter how much diabetes I’ve got (Patient 10).

### Perceptions of disease

Emotional aspects rather than medical tasks dominated the patients’ experiences of chronic diseases. Most patients insisted on leading a normal life and not letting disease take up too much space. Some patients actively focused on the positive sides of life despite significant symptoms, but sometimes symptoms or the fear of serious complications overruled the intended way of living. Most patients were pessimistic about the future.

#### Coming to terms or struggling with disease

Patients did not generally distinguish diabetes from other chronic conditions. Instead, they focused on symptoms that added up to a whole package of disease. To many patients, their first concerns at the time of diagnosis had diminished as they had resigned themselves to the diseases and the subsequent limitations. They had learned to live with their diseases and accepted that the increasing range of complications and forthcoming death were broadly out of their hands

It is just like a path you have to follow completely. I just say: that’s the way things are. I resign myself to this, but I am also informed of how it may develop (Patient 1).

For some patients, the symptoms and the treatment of the chronic diseases caused daily annoyance, and they had continuous difficulties with implementing life style changes and taking medication. The appearance of new complications or diseases could cause a shift to this perspective.

To me, my diabetes is extraordinarily negative. It has changed my life in a drastic way… I have to pay too much attention for myself and remember what I do (Patient 13).

#### Fearing the next complication

Many patients had experienced severe complications like amputation or intensive hospitalization and now feared what would happen next. These patients were aware of living close to new severe complications and even death. The complications seemed to cause more concerns than the fear of dying, illustrated by this patient, who had received intensive care due to acute septicaemia.

I am sick and tired of still thinking of this urinary tract infection - if it returns with the septicaemia. It just came without any warning… Now I understand why the ambulance that picked me up had blue lights and the sirens on (Patient 8).

#### The small bright spots

When coming to terms with advanced chronic conditions and severe complications, there was often a need to focus on the bright spots of their disease, e.g. an improved blood test value or a weight loss. Instead of being depressed, one patient was just relieved that the removal of one toe did not involve the other toes.

In relation to diabetes and my right leg (amputation of the little toe), I actually tend to think: I am happy that it did not develop worse (Patient 12).

#### Being done with the future

Despite many patients having a positive approach to their everyday lives, all patients’ thoughts of the long-term outcomes of disease were characterized by uncertainty and powerlessness. Some had a ray of hope of improved treatments or just hoped for a stabilization of their diseases. However, the experience of a worsening of their conditions and only a little, if any, improvement in their symptoms led to pessimistic views on the long-term future. Some patients worried more about the future of their relatives.

The future is not worth a damn. I am done with the future. I have so much to deal with; the heart and the diabetes… Some days, when I cannot breathe, nothing matters to me, but anyway, I hold on to life because of my children and my grandchild (Patient 11).

### Perceptions of the GP’s support of self-care

Despite differing opinions of self-care, most patients said that their GP played an important role in supporting self-care, but the role varied broadly.

#### The GP as a companion or a guardian

The majority of patients valued the long-term relationship with their GP as a support in dealing with their chronic diseases. The GP’s prior knowledge of the medical records, social context, personal values, and preferences increased mutual confidence and gave some patients a firm basis in the experience of an otherwise fragmented health system. The GP also helped patients to have a useful overview of the uncertainty and complexity related to concurrent chronic diseases. Many patients found support in being listened to and understood by their GP. Some valued talking to the GP about issues other than health, e.g. personal relationships or just conversation.

I do not consult other doctors (in the surgery)… I like the other doctors, but I think something else plays a part here: my doctor knows exactly what I deal with. She knows the full picture (Patient 7).

Some patients experienced the support from their GP as protective; the scheduled consultations about chronic diseases were seen as health checks, where a message of an unchanged treatment was interpreted as an approval of life style. The GPs were also seen by some patients as taking proper action in cases of acute worsening of symptoms, e.g. by introducing new treatments, or making a referral to hospital, or by reassuring patients that symptoms were not signs of serious disease.

#### The GP as a health consultant

Some patients viewed information provided by the GP as their support, and this was less dependent on the relational continuity. The GP could explain the rational basis of the treatment or refer patients to relevant health professionals, such as a dietitian, chiropodist, or community based diabetes education. Some patients avoided health information because they did not want to get confused, while other patients wanted the GP to reduce confusion about contradictory information about diet or medication. Some patients felt a need for the GP to give them a gentle reprimand to trigger life style changes when blood test results were going the wrong way or when insulin looked like the next step in the treatment.

The doctor said: ‘if your blood sugar values have not decreased in three months, you will have insulin prescribed’… so she frightened me, and funny thing you know, I suddenly could change eating habits… saying I am not going to prick myself with a bloody needle (Patient 11).

#### The GP has no supportive role

A few patients were dissatisfied with their GP following a conflict about, e.g. referral or a disease regimen. These patients had lost faith in their GP, and considered finding another. They preferred consulting the practice nurse or other health professionals.

I don’t think that the GP can support me in following the treatment… Obviously, he did not know what to do with the diabetes – otherwise he would not have referred me to the outpatient clinic (Patient 5).

Some patients stated that in the end, it only depended on the patients themselves to follow the recommended treatment, because self-care takes place outside general practice.

The doctors just can’t do anything but talking… It is up to you to obey what they are saying or if you will await a couple of years to be worse off (Patient 6).

## Discussion

### Statement of principal findings

In our study, the patients’ self-care routines that increased well-being and the emotional aspects of chronic disease often compromised the recommended self-care as defined by the GPs. All patients were familiar with these recommendations and already did what they could to adhere to them. Most of the reasons for non-adherence were due to other priorities such as inveterate habits or preferences, situational context, or concurrent diseases. Combinations of somatic and mental diseases could lead to conscious trade-offs in self-care at the expense of the somatic disease, due to the burden of mental symptoms.

The emotional aspects of disease were very prominent in our patients and could shift over time. Despite disabling symptoms and fear of worsening conditions, many patients came to terms with their disease burden and focused on the bright and humorous sides of their lives whenever possible. At other times, however, patients focused on their limitations and found symptoms and treatment a daily struggle.

Most patients found support in their ongoing relationship with the same GP. Some patients valued their GP as a guardian or health consultant or they found that the GP was a catalyst for lifestyle changes. However, some patients stated that self-care depended solely on themselves and a few patients even avoided their GP.

### Strengths and weaknesses of the study

Our study design depended on GPs to identify patients with impaired self-care and this is exactly what DMPs expect GPs to do when managing patients with chronic diseases. Although patients were interviewed only once, their experiences of chronic diseases over time were explored to elicit shifts in symptoms and situational context. We chose to set our study in an economically disadvantaged area due to a high prevalence of multimorbidity but we might also have increased the prevalence of psychosocial problems in our GP-identified patient participants. Despite the fact that the researcher who conducted the interviews with patients is also a GP, we believe we achieved diversity in the expressed opinions about health professionals. We were aware of the risk of conceptual blindness [20], and therefore included health professionals from outside general practice in all phases of the study in an attempt to reduce this risk [21].

### Discussion of results

A key finding was that many patients found their recommended medical regimen difficult to follow if other diseases flared up or social and emotional challenges took their attention and resources away from disease. In accordance with the shifting perspectives model of chronic illness, patient perspectives on disease were cyclic and influenced by factors such as social context and life events [19]. The ability of self-care could affect disease perspective: if a patient had difficulty in adopting a recommended life style change, it could threaten the sense of control. Such a threat could foster a shift in perspective from wellness to illness as our participants experienced when symptoms worsened or challenges in the situational context occurred. Many patients said that they had come to terms with their diseases over time, if symptoms were stable, and in this way the wellness perspective was maintained. From the wellness perspective, self-care actions were perceived as a means to distance oneself from disease, and to perceive oneself as living a normal life.

In this study, symptoms and attention to severe mental disorders seemed to dominate somatic diseases and thereby brought an additional perspective to the foreground. While symptoms of concurrent somatic disease were undoubtedly there, patients consciously viewed symptoms of mental disorders as independent and more present. This suggested that the shifting perspectives model of chronic Illness model could be modified for patients with concurrent mental disorders (Figure 1(B)).

The predominance of the emotional aspects of disease might surprise. Our participants were selected on the basis that they had difficulty following treatment and the public agenda of multimorbidity usually focuses on multiple medications, fragmented health care, and contradictory lifestyle interventions [22]. A recent systematic qualitative review of patients’ challenges in managing multiple chronic diseases showed they reported difficulties in dealing with physical and emotional symptoms, but did not complain about a lack of skill to manage medical tasks [23]. In our study, some patients also had more existential thoughts about life and death. At the same time, they had a general desire to live a normal life with wellness in the foreground beyond an immediate context of multiple diseases. A newly published systematic review of lived experience of multimorbidity found that ‘everyday life work’ such as housework or paid employment had crucial and symbolic significance to evidence the work of being normal [24].

In our interviews, most patients expressed their perceptions through stories. Listening to and interpreting patients’ stories, or narratives, is the essence of narrative medicine, that offers clinicians access to how patients conceptualize disease, how they choose to treat it, and how they respond emotionally to its presence [25]. Patients’ self-care ability is interwoven with previous experiences, which was illustrated by a Norwegian qualitative study of participants in a lifestyle intervention, who could be stuck in old habits due to emotional distress from life baggage [26]. Narrative medicine is a way for clinicians to understand patients’ perspectives of disease and self-care, which also reveal and eventually redefine patients’ emotional responses [27]. One way for doctors to let narratives into a patient-centred interview is by picking up patients’ cues, which allows the patient to turn a simple interaction into a rich unique mosaic of the patient’s current situation in life [28].

Our participants said that their GP could support self-care in different ways, which might reflect individual differences between patients’ needs, preferences and perspectives. Living with a chronic disease involved shifts in perspective within a patient over time. These could also foster a shift in the relationship with the GP [19], but individual character differences between GPs could also be a reason: some patients favoured a rather paternalistic GP, while others preferred a more equal relationship [29]. Meanwhile, the DMPs seem to underestimate the importance of relational continuity [30] by presuming that patients mostly use the GP as a health consultant. The DMPs’ disease-centred model of self-care separated from other aspects of life, reflects this position [11]. Previous research showed that patients value continuity in the doctor-patient relationship more highly than health professionals involved in chronic care [31], and some patients with complex chronic diseases were even reluctant to visit their general practice if they did not see the same GP [22].

We found that patients and GPs perceived self-care differently: patients had found personal self-care routines that increased wellbeing; but GPs had assessed the patients’ self-care ability as impaired and therefore these routines might be inappropriate in the context of a medical regimen. This illustrates the sensitive moral complexities which may emerge when self-care is on the medical agenda [7] and could explain why GPs and patients experience tensions when addressing self-care topics during consultations [4].

According to the DMPs, GPs’ assessment of patients’ self-care ability should determine where and how the treatment of chronic conditions, such as T2DM, takes place. Therefore, the patient perspective is particularly important to include in this assessment, especially in patients with concurrent chronic diseases who often have complex, psychosocial challenges as well. For these patients, deviations from available guidelines are common, and their priorities tend to fluctuate more. Paradoxically, our study showed that patients with a GP-assessed impaired self-care ability felt that they took responsible care of their diseases and believed that the challenges of self-care were largely out of their hands. This gap of self-care perceptions between patients and GPs is important to investigate further. This paper also highlights the need to explore GPs’ clinical experiences with the DMPs, especially regarding self-care, which seem difficult for GPs to use in a clinical setting without adaptations, particularly for patients with multiple chronic diseases.

In the light of this study, the DMPs seem to underestimate fluctuations in self-care ability. The fluctuations are conditioned by the shifting perspectives and priorities, which tended to shift more often in patients with multiple and complex chronic diseases [32]. While the Danish DMPs expect GPs to actively include self-care assessments in chronic care [2], the DMPs themselves have a rather biomedical approach to self-care, which contrast with patients’ perceptions of chronic diseases [11] and the comprehensive definition of self-care as presented in this paper (Box 1). Similarly, a study of how Norwegian GPs experienced applying multiple guidelines found that ‘the map and the terrain did not match’, which could remove focus from the patient [33]. In this sense, our findings of how patients’ life experiences influence perceptions of disease and self-care support the bio-psycho-social disease model and the patient-centred clinical approach as the theoretical basis of being a GP [34]. Regardless of DMPs, the patient’s agenda should still be the starting point for the GP in chronic care to understand and strengthen the resources of the individual patient to improve health [35].

## Conclusion

In this study, we found a gap between GP-assessed impaired self-care and patients’ experiences of managing disease as well as possible. Emotional aspects of patients’ disease perceptions prevailed and could shift between focus on disease or wellbeing over time. Patients regarded GPs’ support of self-care differently, but largely dependent upon an ongoing doctor-patient relationship. Therefore, relational continuity [30] is particularly crucial for patients with complex and concurrent chronic conditions, extensive medical records, and frequent appointments in the health care system.
